# High-Sensitivity Dual-Probe Detection of Urinary miR-141 in Cancer Patients via a Modified Screen-Printed Carbon Electrode-Based Electrochemical Biosensor

**DOI:** 10.3390/s21093183

**Published:** 2021-05-03

**Authors:** Wai-Hung Leung, Chi-Chia Pang, Sow-Neng Pang, Sheng-Xiang Weng, Yu-Lun Lin, Yueh-Er Chiou, See-Tong Pang, Wen-Hui Weng

**Affiliations:** 1 Division of Colorectal Surgery, Department of Surgery, Mackay Memorial Hospital, Taipei City 104, Taiwan; leungwh22@gmail.com; 2 Department of Chemical Engineering and Biotechnology, Graduate Institute of Biochemical and Biomedical Engineering, National Taipei University of Technology, Taipei City 106, Taiwan; benny830205@gmail.com (C.-C.P.); q87076@hotmail.com (S.-X.W.); 11xc11860324@gmail.com (Y.-L.L.); 3 Department of General Medicine, Mater Misericordiae University Hospital, 351402 Dublin, Ireland; benpang2004@gmail.com; 4 Department of Nursing, College of Medicine, Fu Jen Catholic University, New Taipei City 242, Taiwan; 031051@mail.fju.edu.tw; 5 Division of Urology, Department of Surgery, Chang Gung Memorial Hospital, Taoyuan 333, Taiwan

**Keywords:** urine microRNA, miR-141, biosensors, cancer

## Abstract

The screening and diagnosis of cancer are hallmarks of medicine in the aging population. Recently, microRNAs have shown potential for use as biomarkers, which could advance the field of diagnostics. The presence of miRNA-141 in the serum has been well described in several malignancies. However, the invasive approach used for sampling represents the major limitation for its practical application and, hence, its notable absence as a method for screening the general population. In light of this, we aimed to develop a high-sensitivity microRNA (miR) biosensor for application in the diagnosis of all miR-141-associated cancers, such as colorectal cancer (CRC) and breast cancer (BC). The novelty lies in our dual-probe design, which is reliant on the hybridization of the fluorescein isothiocyanate (FITC) targeting probe onto an existing sample of urinary miR-141 in the first step, followed by complementary binding with a biotinylated probe that has been coated on a modified screen-printed carbon electrode (SPCE). The hybridization of the probe and sensor produces signals via the catalytic reduction of H_2_O_2_ at HRP-modified SPCEs in the presence of H_2_O, which was measured by either cyclic voltammetry or chronoamperometry (CA) currents. In our study, the detection and expression of miR-141 in a cohort of colorectal cancer (*n* = 6) and breast cancer (*n* = 4) samples showed that its levels were significantly higher than in a healthy cohort (*n* = 9) (*p* < 0.004). Moreover, our miR sensor demonstrated high stability, reliability, and sensitivity (*p* < 0.0001). This work hopefully provides new information for the detection and monitoring of de novo and existing cancers.

## 1. Introduction

Certain microRNAs (miRs), sometimes referred to as “oncomirs”, are widely demonstrated to be strongly correlated with numerous malignancies. MicroRNAs function in RNA silencing through the post-transcriptional regulation of gene expression by base pairing with complementary sequences of their messenger RNAs (mRNAs) [1,2]. In the pathogenesis of cancer, the expression profiles of certain miRNAs show co-expression with disease-causing genes. As such, they have emerged as excellent biomarkers for cancer [3,4,5]. At the time of this study, the expression of more than 400 miRNAs has been identified in various cancers, representing different statuses of tumor progression; these have been associated with the diagnosis of primary tumors, metastasis, recurrence, and even the prediction of prognosis [6].

The remarkable abundance and stability of miRNAs are what allow for reliable and easy harvesting from tissue samples or bodily fluids. This might include blood, plasma, serum, saliva, tears, or even urine and excreta [7]. The features of miRNA are a very short single-stranded molecule, and a single nucleotide sequence of around 18–25 bp [8]. Although rapid decay occurs intracellularly, miRNAs show significant stability within extracellular vesicles, such as microvesicles, exosomes, or apoptotic bodies; some are bound to RNA-binding proteins or lipoprotein complexes to maintain integrity [9,10,11,12,13,14]. Our choice of hsa-miR-141 (miR-141) as the primary miRNA was mainly due to its proven significance in predicting the progression of many tumors, including prostate cancer, colorectal cancer (CRC), lung cancer, and others [15,16,17]. In particular, stage IV CRC has been reported to have a high expression of miR-141, which predicts a poor prognosis for metastatic CRC [18].

Although novel in its purpose, serum miRNA has been the main focus of numerous clinical assays. Traditional methods to detect the quantification and identification of microRNAs rely on real-time quantitative polymerase chain reaction (qPCR), which is widely used and considered a convenient method with high sensitivity. Moreover, miRNA microarray, Northern blotting, in situ hybridization, cloning methods (such as miRNA serial analysis of gene expression), and nanoparticle methods (for example, the use of electrocatalytic nanoparticle tags for high-sensitivity miRNA expression analysis) are also applied in the clinic; however, all of these techniques have their limitations, being labor- and/or time-consuming and costly [19,20,21,22,23]. Only a few studies have evaluated miRNA in the urine, confined to urologic diseases, lung cancer, pancreatic cancer, and liver cancer [16,24]. In contrast to blood or tissue biopsies, urine sampling is completely noninvasive. Furthermore, the safety profile, collection time, and sample quantity are far superior to those of tissue or blood sampling. It is envisioned that, given the ease of collection, self-sampling by patients would be the goal, thereby reducing diagnostic and follow-up intervals. Of course, the limitations of urinary miRNA cannot be neglected. Concentrations of urinary miRNA are known to be extremely low (<0.01 vol%) [25]. Based on these factors, the sensitivity of the biosensor would be critical for determining reliability in clinical settings.

Herein, the novelty of our miRNA sensor is in its design. The probe is a tumor-related miR-141 that harbors a fluorescein isothiocyanate (FITC) dye (denoted as the FITC probe); the FITC probe is complementary to one end of the sample miRNA sequence, while the other end binds to another biotinylated capture probe (denoted as the bioreceptor probe), which is pre-integrated onto the surface of the screen-printed carbon electrode (SPCE). Once the single strand of the sample miRNA sequence has been caught by both probes, the FITC further binds with an anti-fluorescein HRP, which acts as a reporter via a reaction that catalyzes the decomposition of hydrogen peroxide. Via oxidation and reduction, electrons are then picked up through the streptavidin/biotin system on the screen-printed carbon electrode (SPCE). The produced signal is then reported as the chronoamperometry (CA) current, which is correlated with the miR-141 expression level. To the best of our knowledge, this is the first urinary miR-141 biosensor that allows for direct hybridization onto urinary samples of miRNA without initial amplification. In essence, our biosensor follows the elementary concept of the urinary dipstick, similar in its accessibility for sampling as well as its screening efficiency effectiveness.

## 2. Materials and Methods

### 2.1. Reagents and Chemicals

CM-dextran sodium salt (CMD-Na), phosphate-buffered saline (PBS), 1-ethyl-3-(3-dimethylaminopropyl) carbodiimide (EDC), N-hydroxysulfosuccinimide (NHS), ethanolamine, potassium chloride (KCl), potassium ferricyanide (Fe(CN)_6_^3−^), hydrogen peroxide (H_2_O_2_), diethyl pyrocarbonate (DEPC), and 3,3′,5,5′-tetramethylbenzidine (TMB) were purchased from Sigma-Aldrich (Sigma-Aldrich St. Louis, MO, USA). Anti-fluorescein horseradish peroxidase (HRP) is a 40,000 dalton protein supplied by Abcam (Cambridge, UK). MES free acid monohydrate, hydroxymethyl-aminomethane (Tris), and ethylenediaminetetraacetic acid (EDTA) were purchased from Amresco (Amresco Inc., Solon, OH, USA). Core streptavidin was from BiVision (Bioptics, Tucson, AZ, USA). Sodium chloride (NaCl) was from Promega Corporation (Madison, WI, USA). The biotinylated ssDNA probe (bioreceptor probe) (5′-CAGACAGTGTTA-3′-(CH2)_6_-biotin), fluorescein (FITC) ssDNA probe (detector probe) (FITC-(CH2)_6_-5′-CCATCTTTAC-3′), and artificial mimic targeting sequence (5′-UAA CAC UGU CUG GUA AAG AUG G-3′) were designed by us and made by Genomics (Taipei, Taiwan). Screen-printed carbon electrodes (SPCEs) were purchased from Zensor R&D (Taichung, Taiwan).

### 2.2. Methods and Apparatus

#### 2.2.1. Electrochemical Measuring

**Figure 1:**
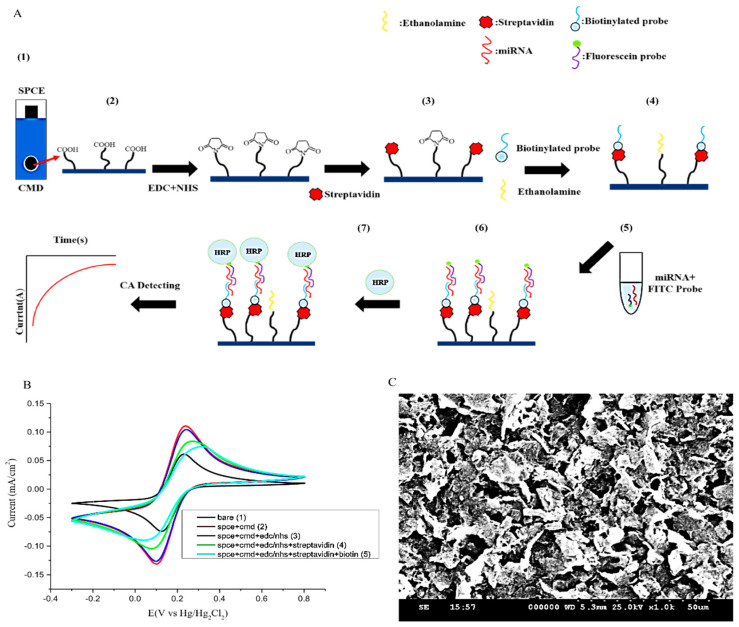
Establishment of the miR biosensor. (**A**) Schematic representation of the fabrication and operation of the miR biosensor, starting with (1) the process of creating the screen-printed carbon electrode (SPCE) for the detection of miR-141. (2) CMD was then used to modify the SPCE to provide the reactive groups, followed by EDC and NHS crosslinking to the electrode. (3) Streptavidin was subsequently added to the electrode. (4) After this, the biotinylated probe was immobilized onto streptavidin, with ethanolamine occupying the remaining SPCE surface. (5) In order to detect the miR-141, pretreatment of miRNA and the fluorescein-modified detection probe (FITC probe) was conducted in a separate medium. (6) The addition of the miR-141/FITC probe complex to the functional SPCE surface allowed for hybridization. (7) With the addition of anti-fluorescein horseradish peroxidase (HRP), a catalyzed redox reaction occurs between tetramethylbenzidine (TMB) and H_2_O_2_ at the surface of the electrode. (**B**) Generating electrochemical signals (CA currents) in every step; without the presence of miR-141, no CA currents would be generated. (**C**) SEM image showing the surface of SPCE after all modification steps.

For electrochemical experiments, the apparatus used in this study was the aMetrohm (Herisau, Switzerland) AutoLab PGSTAT204 electrochemical workstation, and data were further analyzed by NOVA1.11 (Herisau, Switzerland). The counterelectrode was made of platinum, a saturated calomel electrode was used as a reference electrode, and the working electrode was a surface-modified SPCE. Two electrolyte formulations were applied for the experiments. First, 0.1 M KCl and 5 mM ferricyanide were used for cyclic voltammetry to analyze the surface resistance. Second, a 0.4 mM TMB and 0.4 mM H_2_O_2_ solution was applied on chronoamperometry (CA) measurement with a setting of −200 mV against a saturated calomel electrode; the scan rate was 0.05 V s^−1^ and the electroreduction current was measured at 200 s after the HRP redox reaction reached steady state. The data analysis was performed using Origin (Northampton, MA, USA) 9.0, and the statistical analysis was performed using GraphPad Prism 8 (GraphPad Software, La Jolla, CA, USA) (Figure 1B).

#### 2.2.2. FITC Probe and Bioreceptor Probe Design

The goal of the miR biosensor was to enhance the signal and increase sequence targeting. In our study, the miR biosensor consisted of two major parts derived from the miR-141 sequence, divided into two sections. One is a biotinylated capture probe, which is linked onto the SPCE surface and denotes the bioreceptor probe (5′-CAGACAGTGTTA-3′-(CH_2_)_6_-biotin). The remaining part of the sequence is denoted as the FITC probe (FITC-(CH_2_)_6_-5′-CCATCTTTAC-3′), whose function is to attach to the miR-141 in urine. The FITC fluorescein dye is used to produce a signal once the reaction with HRP occurs. The hybridization of the two probes onto the target miR-141 single nucleotide allows the transducer to convert signals from the bioreceptor‒microRNA interaction into measurable signals via the AutoLab PGSTAT204 electrochemical workstation (Figure 1).

#### 2.2.3. Modification of SPCE

In order to crosslink the bioreceptor probes onto the surface of the SPCE, modification of the SPCE was necessary. Initially, 50 μL carboxymethyldextran sodium salt (CMD-Na) (50 mg/mL) was used to saturate the work surface for 16 h to create a carboxylic (COOH) functional group, with further activation with a mixture of 8 mg/mL 1-ethyl-3-(3-dimethylaminepropyl) carbodiimide (EDC) and 22 mg/mL N-hydroxysulfosuccinimide (NHS) in 0.1 M MES buffer (pH 4.7) for 15 min at room temperature. This allowed the leading streptavidin (BioVision Inc. Mountain View, CA, USA) to conjugate on the surface. The last to be conjugated is the 5 μM biotinylated single-strand oligonucleotide sequence (ssDNA) probe that contains the partially mimicked miR-141 sequence. This forms the functional surface of the “bioreceptor” that complements the corresponding end sequence of the target miR-141. Ethanolamine (1 M) was used to block the remaining activated sites. All experimental solutions were configured using 0.1% DEPC-treated water to inactivate RNase and ensure the stability of reactions. Thus, SPCE was functionalized and ready to use (Figure 1A). Scanning electron microscopy (SEM) was then performed to confirm the suitability of the surface of the SPCE after completing all the steps (Figure 1C).

#### 2.2.4. Reactions between Urinary miR-141, the Bioreceptor Probe, and the FITC Probe

There are four main processes before signal detection. The FITC detector probe is initially mixed with miR-141 samples, then instilled with STE buffer (0.1 M sodium chloride, 10 mM Tris buffer, 1 mM EDTA, pH 8.0) at 65 °C for 3 min, and subsequently cooled down to 25 °C. This process marks the completion of the initial preparation to allow miR-141 hybridization with the modified SPCE, which occurs by direct application to the working surface for 15 min at room temperature. After this, 20 μL of anti-fluorescein antibody (HRP) (Abcam) was applied by dripping for 15 min at room temperature to allow for the annealing of HRP onto the sample/FITC probe. Finally, the whole SPCE was dipped into 0.4 mM of 3,3′,5,5′-tetramethylbenzidine (TMB) and 0.4 mM of H_2_O_2_ (Sigma) in a solution of DEPC water mixture to elicit the signal that is measured (Figure 1).

#### 2.2.5. Urine Collection from Cancer Patients

In total, 19 urine samples were obtained for the study. Of these, 10 came from 9 cancer patients (4 breast cancer patients, 5 CRC patients), and all were obtained prior to surgical intervention, with the exception of one individual (sample nos. C1 and C2, collected from the same CRC patient, one presurgery and one postsurgery, at a one-month interval). An equal number of normal individuals were used as controls. This study was carried out with the approval of the Human Subject Research Ethics Committee/Institutional Review Board (IRB:18MMHIS017) of Mackay Memorial Hospital (Taipei, Taiwan).

#### 2.2.6. Urinary microRNA Isolation and Measurement from Clinical Samples

The total RNA and all sizes of exosomal RNAs, including microRNA from urine, were extracted using Norgen’s Urine MicroRNA Purification Kit (#29000, Norgen Biotek, Ontario, ON, Canada). The process was in accordance with the manufacturer’s protocol with minor modifications, while only small RNAs with a molecular size less than 200 nt would be isolated and purified. In brief, 1 mL of urine per sample was lysed and centrifuged at 8000 rpm. Samples were double-washed and centrifuged at 14,000 rpm. The purified miRNA was eluted using 50 μL elution buffer and stored at −80 °C until testing. The final concentration yield was dependent on the specimen; the mean RNA concentrations were around 30 ng/10 μL.

#### 2.2.7. miR-141 Quantitative Measurement in Urine via Quantitative Reverse Transcription PCR (qPCR)

To demonstrate the accuracy of the data produced by the miR biosensor, qPCR of miR-141 was performed in triplicate for each sample as a control for comparison. In brief, after the extraction of RNA, the quantity of extracted RNA was measured using a Multiskan GO spectrophotometer (Thermo Fisher Scientific, Inc. Cleveland, OH, USA), and then a total of 10 ng RNA from each sample was reverse-synthesized to cDNA using a TaqMan MicroRNA Reverse Transcription kit (Thermo Fisher Scientific, Inc.) according to the manufacturer’s protocol. The expression of human miR-141 (hsa-miR-141) was quantified through qPCR using a TaqMan MicroRNA Assay kit (Thermo Fisher Scientific, Inc.) and an Applied Biosystems Veriti Thermal Cycler (Thermo Fisher Scientific, Inc.) according to the manufacturer’s protocols. The thermocycling conditions used were as follows: 10 min at 95 °C, 15 s at 95 °C, and 60 s at 60 °C for 40 cycles. The following primers were used: hsa-miR-141, Mature miRNA Sequence: UAACACUGUCUGGUAAAGAUGG. Furthermore, the average of 10 normal subjects was normalized to 1 and used as a baseline for further comparison.

#### 2.2.8. Statistical Method

Graph analysis was conducted using Origin 9.0, and the statistical analysis was performed using GraphPad Prism version 8 to compare test and control samples and calculate *p*-values.

## 3. Results and Discussion

Regarding the development of nucleotide biosensors, several types of devices have recently been reported, as shown in Table 1.

In [26,27,28,29], for example, using carbon nanomaterial-based electrochemical biosensors, the detector exhibited a low response in the 40‒110 nM range with a limit of detection of 20 nM [30]. Furthermore, MnO_2_NFs/NG was found to have remarkable electrocatalytic activity toward dopamine, and uric acid might give valuable insights into new nanomaterials to be applied in the development of biosensors [31]. Detecting miRNAs in urine remains a challenge with conventional methods, such as qPCR, due to the limited number of miRNAs in urine, the time-consuming nature of the assays, and fluorescence decay requiring technical skill and materials. We evaluated the different miR-related detection methods for comparison (Table 1). The novelty of the designed miR biosensor is the use of a dual probe that hybridizes to urinary miR-141 to increase the accuracy of detection; however, all the components are necessary in order to attain the necessary sensitivity and selectivity for clinical applications, including the stable bioreceptor probe binding on the SPCE surface, the affinity of the probes, the hybridization of both the bioreceptor probe and the FITC probe to the sample miRNA sequences, and the concentration of urine miRNA. The results were recorded in CA measurements and compared with the three tests.

**Figure 2:**
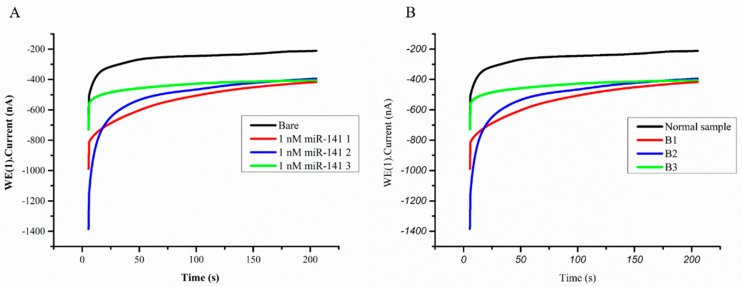
The stability of the functionalized screen-printed carbon electrode (SPCE)-based electrochemical miR biosensor. (**A**) Triplicate test with 1 nM of mimic miR-141 to check current response and stability. (**B**) SPCE tested on three breast cancer samples (B1‒B3) and mean of normal samples.

The minimum and maximum currents from the bare sample to 1 nM of mimic miR-141 were 138 and 400 nA, respectively (Figure 2A), corresponding to a range of urinary miR-141 concentrations between 10^−8^ and 10^−12^ M (Figure S1) (see Supplementary Material). qPCR was employed as a reference for accuracy, which showed the consistency of the miR biosensor. In comparison with the control cases, samples derived from CRC and BC patients presented results that were statistically significant at *p* < 0.0001 and 0.0076, respectively.

### 3.1. Optimization of Experimental Variables

**Figure 3:**
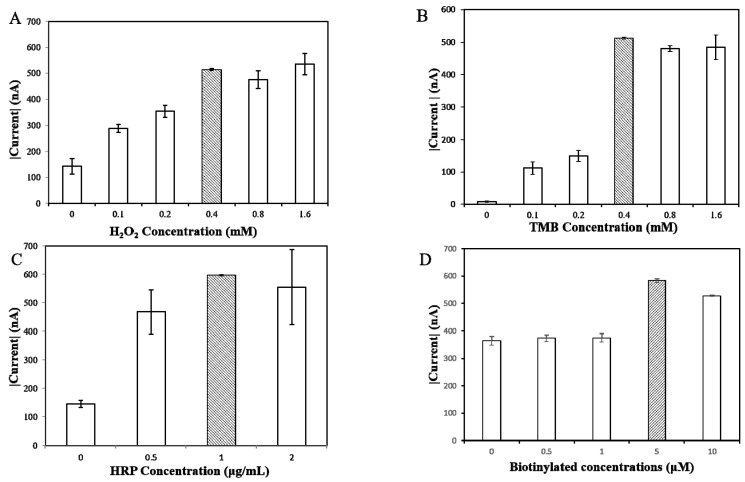
Optimal concentrations of reagents tested on the miR biosensor, shown as the shaded bar. (**A**) H_2_O_2_; (**B**) 3,3′,5,5′-tetramethylbenzidine (TMB); (**C**) anti-fluorescein HRP; (**D**) biotinylated probe concentration.

The generation of signals is derived from HRP, catalyzing the transfer of two electrons from the TMB substrate to the H_2_O_2_, producing H_2_O and an oxidized TMB substrate. The signals produced via the oxidized TMB substrate are measured in CA currents, as shown in Figure 3. In order to optimize experimental values, different H_2_O_2_ concentrations (0, 0.1, 0.2, 0.4, 0.8, 1.6 mM) were trialed against a 2 mM concentration of TMB substrate. As shown in Figure 3A, 0.4 mM of H_2_O_2_ produced the strongest and most consistent CA current; through the same process, 0.4 mM of TMB substrate was found to be the optimal concentration (Figure 3B); again, from a grading concentration of 0, 0.5, 1, 2 μg/mL, HRP concentrations of 1 μg/mL showed the highest current response (Figure 3C). The ideal conditions for the further evaluation of miRNAs were therefore determined to be 0.4 mM of H_2_O_2_, 0.4 mM of TMB substrate, and 1 μg/mL of HRP.

The density of the biotinylated probe on the surface of the SPCE was another factor that determined the efficiency of hybridization onto the targeted sequences. In an effort to optimize the density in correlation to the signal response, variable concentrations of biotinylated capture probes (0.1, 0.5, 1, 5, 10 μM) were added to 10 nM of miR-141 sequences. A concentration of 0.5 μM demonstrated the highest current response, as shown in Figure 3D.

As mentioned above, in order to conjugate miR-141 sequences to our miR biosensor, FITC probe hybridization with miR-141 sequences is necessary. In this study, different concentrations of FITC probes (0.1, 0.5, 1, 2.5, 5 μM) were added to 10 nM of sample miR-141, which showed the highest current response at 1 μM (Figure S2).

### 3.2. Sensitivity and Reliability of the miR Biosensor

The reliability of the miR biosensor after optimization of the experimental conditions was defined by the comparison of CA current between normal and miR-141-positive samples. The minimum and maximum currents measured in miR-141-positive samples were 138 and 362 nA, respectively. Control samples with no miR-141 presence had a range from 95 to 183 nA (Figure 2). As shown, the maximum current from the miR-141-positive samples was double that of the control samples. We recognized the overlap of higher-amplitude currents of control samples and the lower amplitudes of miR-141 samples. A sensitivity evaluation of the miR biosensor was then carried out using artificially synthesized sequences of miR-141, with test concentrations ranging from 10^−8^ to 10^−12^ M. The results were close to exact linearity on the calibration plot (*R*^2^ = 0.9951) (Figure S1). We further compared the limitation of detection (LOD) for similar sensor devices previously developed for miR-141 detection, which applied serum samples and where sensor detection was based on target-induced fluorescence enhancement and PicoGreen. The detection limits of the biosensor for miR-141 were 70 and 113.8 nmol L^−1^ in deionized water and serum samples, respectively [32].

### 3.3. Stability and Repeatability of the miR Biosensor

During the study, we observed a CA current signal reduction from the miR biosensor over time. The rate of decay was gradual, and the most significant difference in our results was on day 5, when there was a more noticeable drop in the current signal. The measured current signals decreased by 20% on the seventh day (Figure S1). This would denote the least acceptable signal strength for accurate detection of miR-141. To assess repeatability, a substrate of 1 nM of a mimic artificial miR-141 sequence was tested three times, which gave consistent current responses at 500 nA. The results were compared with signals with no target sequence capture at 100 nA (Figure 2A). The same method was applied to clinical samples, using three breast cancer samples and a mixture control sample; the results ranged from 500 to 600 nA in cancer cases, compared with 300 nA (Figure 2B).

### 3.4. Sensitivity and Selectivity of miR Biosensor in Cancer Patients’ Urine

In clinical urine samples, miR-141 is not the only sequence of miR present. To actuate the concentration of urine miR-141, qPCR was employed to demonstrate the selectivity and sensitivity of the miR biosensor in this study.

**Figure 4:**
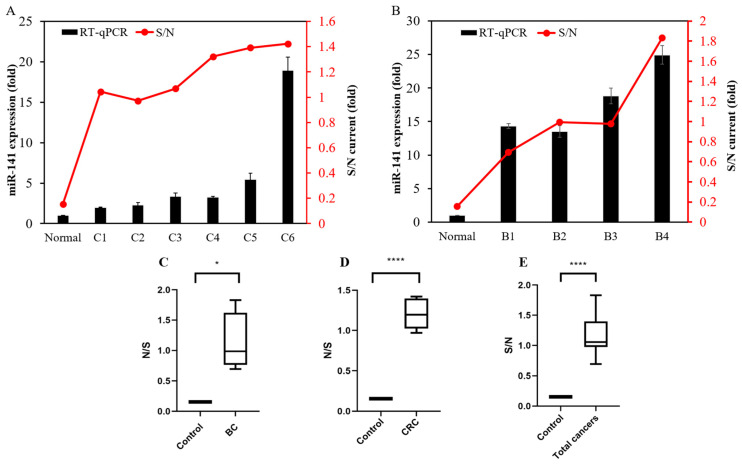
The miR-141 biosensor specificity (red lines) was confirmed by the ratio of total RNA in cancer patient urine samples to the mean of normal urine samples. qPCR (black bars) analysis was used to confirm miR-141 biosensor results, which shows consistency for the (**A**) 6 colorectal cancer (CRC) urine samples and (**B**) 4 breast cancer (BC) urine samples. Box plots and GraphPad Prism 8.2.1 were used to carry out nonparametric data analysis and assess data for descriptive statistics, demonstrating significant differences between miR-141 in cancer patient urine and normal urine as a cohort. (**C**) CRC cases, *p*-value 0.0213; (**D**) BC cases, *p*-value 0.0076; (**E**) total cancer cases, *p*-value 0.00000072.

Herein, we screened 10 normal cases: 6 colon cancer and 4 breast cancer cases. Both normal sample and cancer sample expressions of miR-141 were determined with the miR biosensor, with qPCR used for comparison (Table 2). Results of expression were recorded in ratios, with the control group or the normal samples being the denominator. As expected, both qPCR and the miR biosensor showed at least double the expression in cancer samples compared with normal samples. Moreover, the results of the miR biosensor showed consistency with the results of qPCR (Figure 4A,B).

Subgroup and combined results of CRC and BC patients were then compared against the control samples. The significance of the results is expressed in ratios via box plots. Overall CA currents were, on average, 10 times higher in cancer samples compared with the control (*p* < 0.0001), 12 times higher in colon cancer samples compared with the control (*p* < 0.0001), and 10 times higher in breast cancer samples compared with the control (*p* < 0.0076) (Figure 4C‒E, Table 2). To the best of our knowledge, a high expression of miR-141 is commonly observed in circulating blood and tumor tissues, but there have been no reports of miR-141 expression in urine from breast cancer and colorectal cancer patients, indicating the significant novelty of our findings.

An interesting observation was made during this study. In one patient with CRC who gave two separate urine samples (C1 and C2), before and after recurrent liver metastasis surgery, the miR-141 expression data from the qPCR assay did not show significant differences, whereas the miR biosensor indicated a slight decrease in the current (Figure 2A). However, this is only speculation, given the rudimentary evidence. Nevertheless, if we can find more robust evidence, this could also become a new method for tumor progression surveillance.

A second observation can be made that a significantly higher density of miR-141, as expressed by PCR, does not indicate the same proportion of signal increase produced by the miR biosensor. The limit of detection (LOD) depends on the density of the surface probes, which has a large effect on the efficiency of hybridization. The more modifications there are on the probe, the higher the negative charge it carries on the surface, leading to the electrostatic repulsion of the target miR-141 sequence. Furthermore, the high density of the detected probe also leads to a reduction in the space in which the target sequence can hybridize, making hybridization more difficult.

## 4. Conclusions

This work presents a novel amperometric bioplatform with which to test for the presence of miR-141. Through the optimization of the miR-141 biosensor, a distinct difference in signal can be measured between the urine samples of normal individuals and cancer patients. The data indicated significant differences, which suggests that this is a novel method for the detection of urine miR-141 in colorectal and breast cancers. Being noninvasive and easily repeatable, yet simple in concept, the studied biosensor paves the way for new cancer diagnostics and surveillance.

## Figures and Tables

**Table 1:** Comparison of miRNA detection-related biosensors.

Type of Sensor	Base of Platform	Limitation of Detection (LOD)	References
Electrochemical sensor	SPCE	0.1 pM	Current study
Photoelectrochemical sensor	Photoactive materials	3.3 aM	[26]
Surface plasmon resonance biosensor	SPR sensorchips	0.045 pM	[27]
Surface-enhanced Raman spectroscopy optical nanosensor	SERS-active substrates	0.083 fM	[28]
Fluorescent biosensor	Nanomaterials	0.1 fM	[29]

**Table 2:** The expression level of miR-141 detected in colorectal and breast cancer patients’ urine by using RT-qPCR.

Case Number	Fold
Normal (as a basal line)	1
Colorectal cancer	-
C1	2.014667
C2	2.281667
C3	3.367333
C4	3.367333
C5	5.472667
C6	18.94367
Breast cancer	-
B1	14.318
B2	13.49367
B3	18.8235
B4	24.94014

C represents a colorectal cancer case; B represents a breast cancer case.

## Supplementary Materials

The following are available online at https://www.mdpi.com/article/10.3390/s21093183/s1, Figure S1: Calibration plot of miR-141 concentration and CA current response. The corresponding CA current response with miR-141 concentration, ranging from 10–8 M to10–12 M, producing R^2^ = 0.9951, Figure S2: Current reduction over time measured in percentage (denominator being day 0 current response). A steady decline of current response over a seven-day period, maintaining 80% of its original capacity and accuracy.
