# Supplementary material for: High-Sensitivity Dual-Probe Detection of Urinary miR-141 in Cancer Patients via a Modified Screen-Printed Carbon Electrode-Based Electrochemical Biosensor
Source: Sensors (Basel). 2021 May 3;21(9):3183. doi: 10.3390/s21093183 (PMC8125155; doi:10.3390/s21093183)
Supplement: Supplementary file 1 [file sensors-21-03183-s001.zip › sensors-1169771-supplementary.pdf]

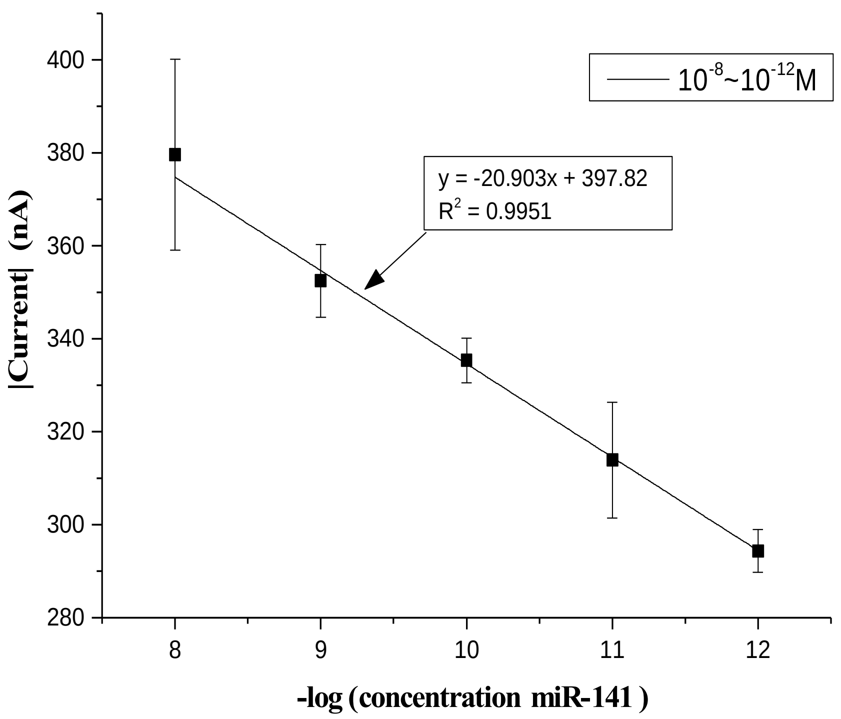

Figure S1. Calibration plot of miR-141 concentration and CA current response. The corresponding CA current response with miR-141 concentration, ranging from10^{-8}M to10^{-12}M, producing R^2 =0.9951.

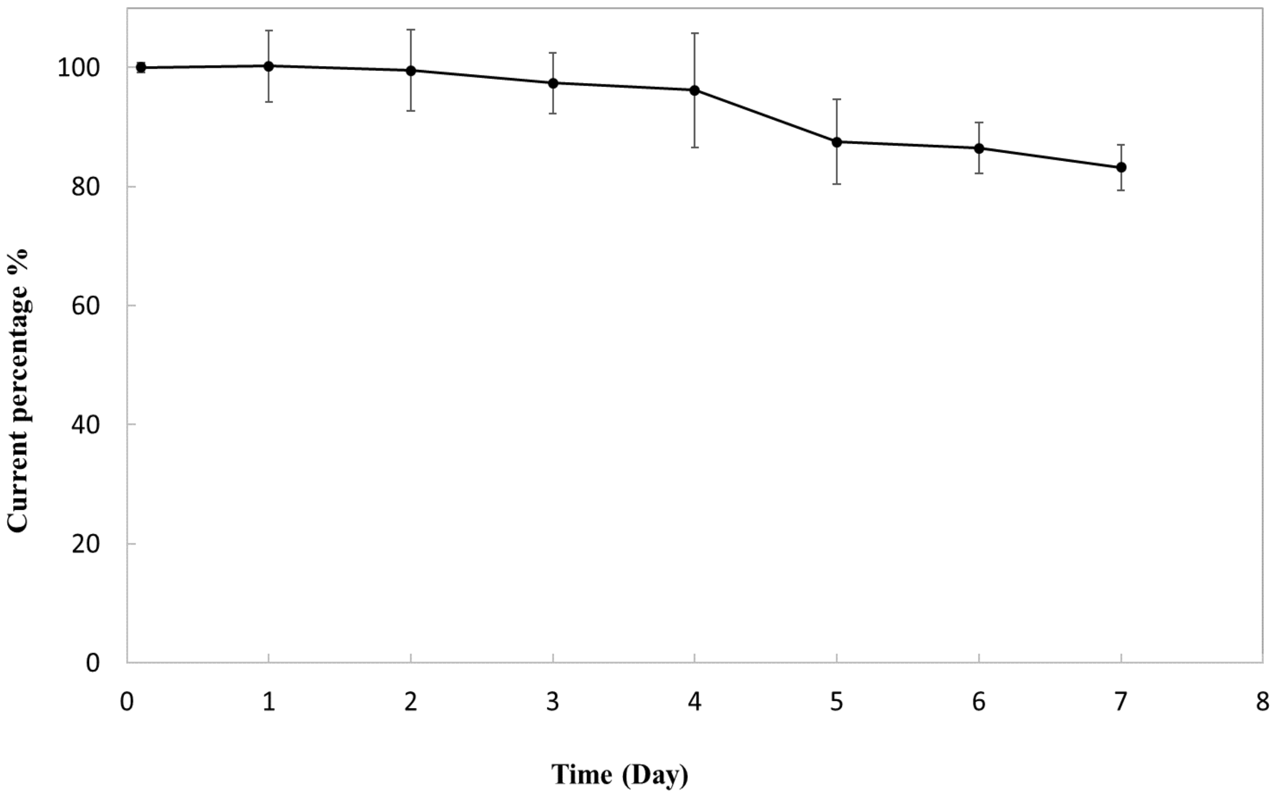

Figure S2. Current reduction over time measured in percentage (denominator being day 0 current response). A steady decline of current response over a seven-day period, maintaining 80% of its original capacity and accuracy.
